# The Great Mimicker: Variable Presentations of Xanthogranulomatous Salpingo-Oophoritis in a Case Series of Seven Patients From a Rural Tertiary Care Center

**DOI:** 10.7759/cureus.114481

**Published:** 2026-08-13

**Authors:** Ekta Rani, Saloni Saloni, Sonam Juneja

**Affiliations:** 1 Pathology, Guru Gobind Singh Medical College and Hospital, Faridkot, IND; 2 Pathology, Advanced Cancer Institute, Bathinda, IND

**Keywords:** inflammation, malignancy, mimicker, oophoritis, pathology, salpingitis

## Abstract

Xanthogranulomatous salpingo-oophoritis (XGSO) is an uncommon, non-neoplastic chronic destructive inflammatory condition. Its clinical and radiological features frequently masquerade as advanced ovarian malignancy or refractory pelvic inflammatory disease, posing a notable diagnostic challenge. Histopathological examination remains the gold standard for diagnosis. A retrospective clinicopathological evaluation of seven distinct cases of xanthogranulomatous adnexal inflammation was performed. Clinical symptoms, diagnostic imaging, intraoperative details, surgical approaches, and gross and microscopic evaluations were thoroughly analyzed. The age of the patients varied from 24 to 68 years. The predominant clinical manifestations included lower abdominal pain, fever, palpable mass, or abnormal uterine bleeding. In the majority of cases, imaging modalities primarily revealed complex solid-cystic ovarian masses, mimicking pelvic malignancy. Surgical management ranged from cystectomies to total abdominal hysterectomies with bilateral salpingo-oophorectomy. Histopathology confirmed the diagnosis in all cases, demonstrating replacement of the target adnexal parenchyma by sheets of lipid-laden foamy histiocytes intermixed with plasma cells, lymphocytes, polymorphonuclear leukocytes, occasionally scattered giant cells, and adjacent stromal fibrosis. XGSO remains a true mimic of pelvic malignancies. Histopathological analysis is essential for accurate identification and differentiation from neoplasm or other granulomatous conditions. Clinical awareness of the condition can prevent unnecessary radical surgical overdiagnosis.

## Introduction

Xanthogranulomatous salpingo-oophoritis (XGSO) is a rare, non-neoplastic chronic inflammatory condition that frequently mimics malignancy [1-4]. While xanthogranulomatous inflammation (XGI) commonly affects organs like the gallbladder and kidney, involvement of the ovaries and fallopian tubes is rare [5,6]. XGI often causes extensive destruction of the normal anatomical structure of the affected organs, leading to significant diagnostic pitfalls and clinical challenges [2,5,7].

Histopathologically, XGI is characterized by collections or dense sheets of foamy or pigment-laden macrophages accompanied by acute and/or chronic inflammation [4,8,9]. In this case series, we describe seven cases of XGSO, highlighting their clinical presentations, radiological findings, and subsequent confirmation by histopathological examination. These findings emphasize the highly variable clinical nature of this entity and aim to increase clinical awareness, thus avoiding unnecessary radical surgery and reducing patient morbidity.

## Materials and methods

A retrospective observational analysis of seven cases diagnosed with XGSO was conducted in the Department of Pathology, Guru Gobind Singh Medical College and Hospital, Faridkot, between May 1, 2024, and June 30, 2025. Informed consent was obtained from all patients. All cases diagnosed with XGSO during the defined period were included. Cases in which clinical details or histopathology slides were not available were excluded. Histopathological slides from these cases were retrieved from the departmental archives. Clinical details, including age, parity, presenting complaints, previous surgical and medical history, and associated risk factors, were obtained from the medical records.

Additionally, radiological investigations, tumor markers, and other relevant investigations, such as culture reports, were collected when available. Macroscopic details, including lesion size, gross appearance of the cut surface, site of involvement, laterality, etc., were obtained from the available histopathology reports. Histopathology slides were reviewed by two independent pathologists, and the diagnosis was established based on the characteristic presence of lipid-laden foamy macrophages admixed with inflammatory cells (acute or chronic). Additional microscopic features that were assessed included the presence of multinucleated giant cells, hemosiderin-laden macrophages, and evidence of hemorrhage or necrosis.

## Results

The patients ranged in age from 24 to 68 years. All seven patients were married women, consisting predominantly of sexually active individuals of reproductive age and postmenopausal women. Two cases had a previous history of lower segment cesarean section (LSCS), and one patient had undergone tubal ligation in the past. Comorbidities included hypothyroidism in two cases, diabetes in one case, and obesity in one case. The chief presenting complaint in the majority of cases was abdominal pain. Radiology reported solid-cystic ovarian masses in most cases, with no radiologically visible lesions in any of the fallopian tubes.

Clinical diagnosis

Preoperatively, the clinical and radiological findings in these seven cases yielded a broad differential diagnosis, underscoring this condition’s great mimicker potential. In the majority of cases (Cases 1, 4, and 7), an adnexal or ovarian neoplasm was the primary suspicion due to the presence of complex solid-cystic masses on imaging. Other preoperative differential diagnoses included symptomatic uterine leiomyomas or adenomyosis (Cases 2 and 3), pelvic inflammatory disease (PID)/pelvic abscess (Case 7), endometrioma (Case 6), and benign ovarian cysts (Cases 2 and 5). This wide array of initial clinical impressions underscores how closely XGSO masquerades as both neoplastic and non-neoplastic gynecological conditions, making histopathological analysis essential for confirmation.

Histopathological characteristics

**Figure 1:**
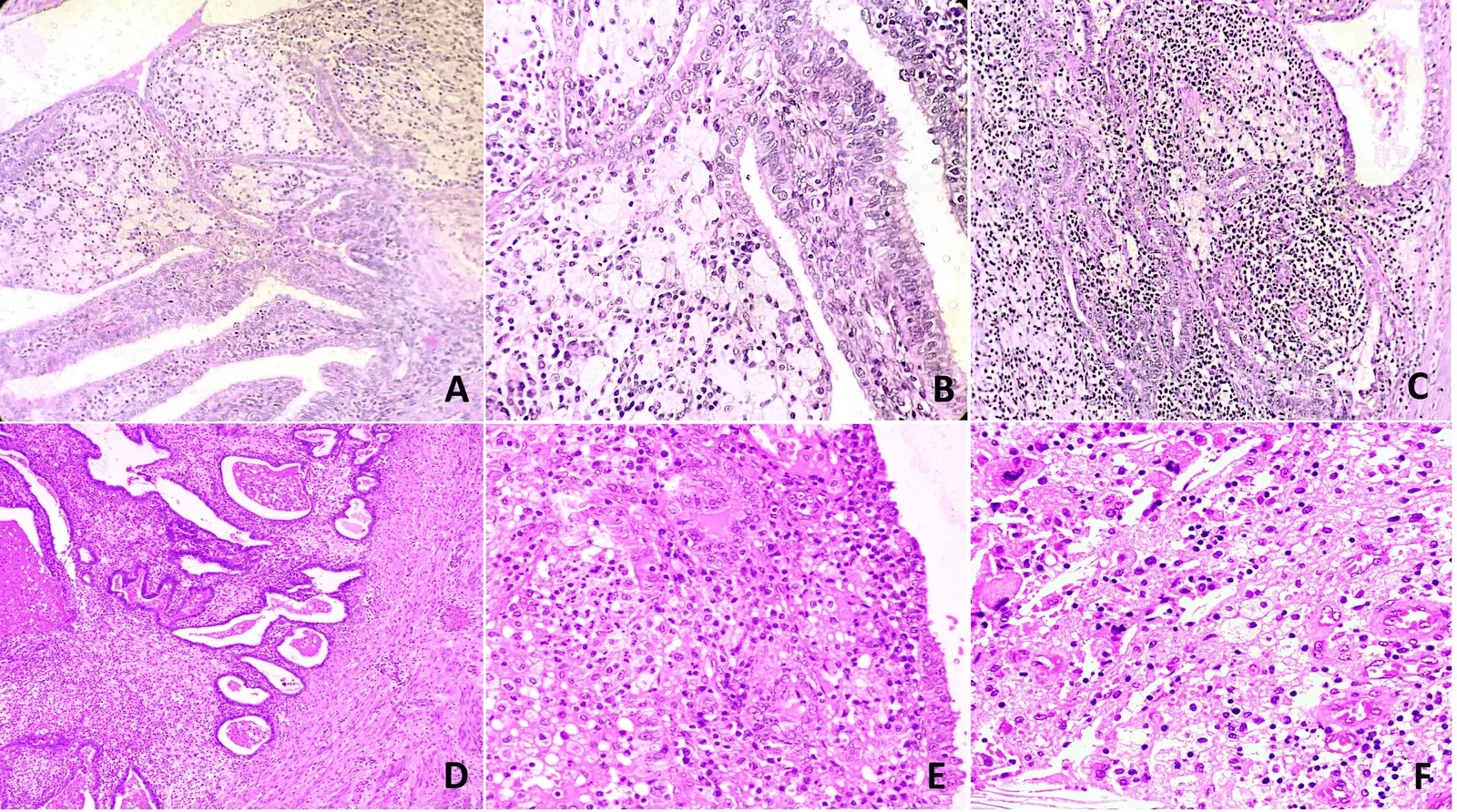
Histopathological spectrum of xanthogranulomatous salpingitis. A-C (Case 2): A. Dense inflammatory infiltrate replacing normal tubal structure (low-power view, 40×, H&E); B. Extensive subepithelial, sheet-like accumulation of lipid-laden foamy macrophages mixed with chronic inflammatory cells (200×, H&E); C. Prominent infiltration of lymphocytes and plasma cells around the plical extensions (100×, H&E); D-E (Case 5): D. Microscopy from a 68-year-old woman with bilateral xanthogranulomatous salpingitis, highlighting marked thickening of the fallopian tube wall due to the inflammatory process (100×, H&E); E. Dense clusters of foamy macrophages along with plasma cells and lymphocytes (200×, H&E); F (Case 6): High-power view showing enlarged histiocytes with abundant, finely vacuolated cytoplasm and eccentrically placed nuclei (400×, H&E).

**Figure 2:**
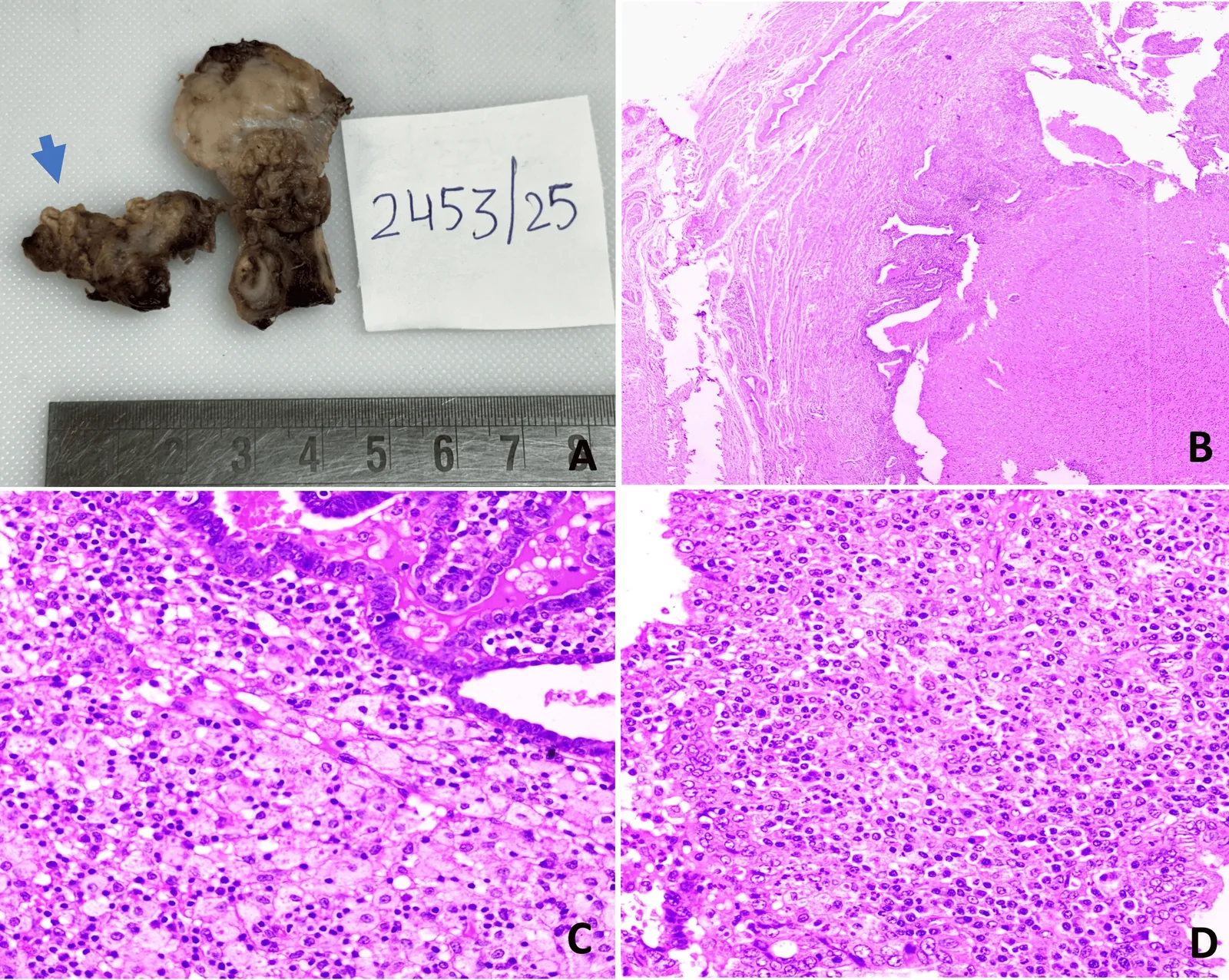
Gross and microscopic features of xanthogranulomatous salpingitis (Case 7). A. Gross photograph showing the ovary with an adjacent, cut-open dilated fallopian tube. The lumen shows gray-yellow areas (arrow); B. Low-power view showing replacement of normal tubal stroma by inflammatory cells. Intraluminal hemorrhage is noted (400×, H&E); C. Sheets of foamy macrophages accompanied by lymphocytes and plasma cells flanking residual plical epithelium (400×, H&E); D. Acute inflammatory infiltrate (neutrophils) in a case of xanthogranulomatous salpingitis along with chronic inflammatory cells and foamy macrophages (400×, H&E).

**Figure 3:**
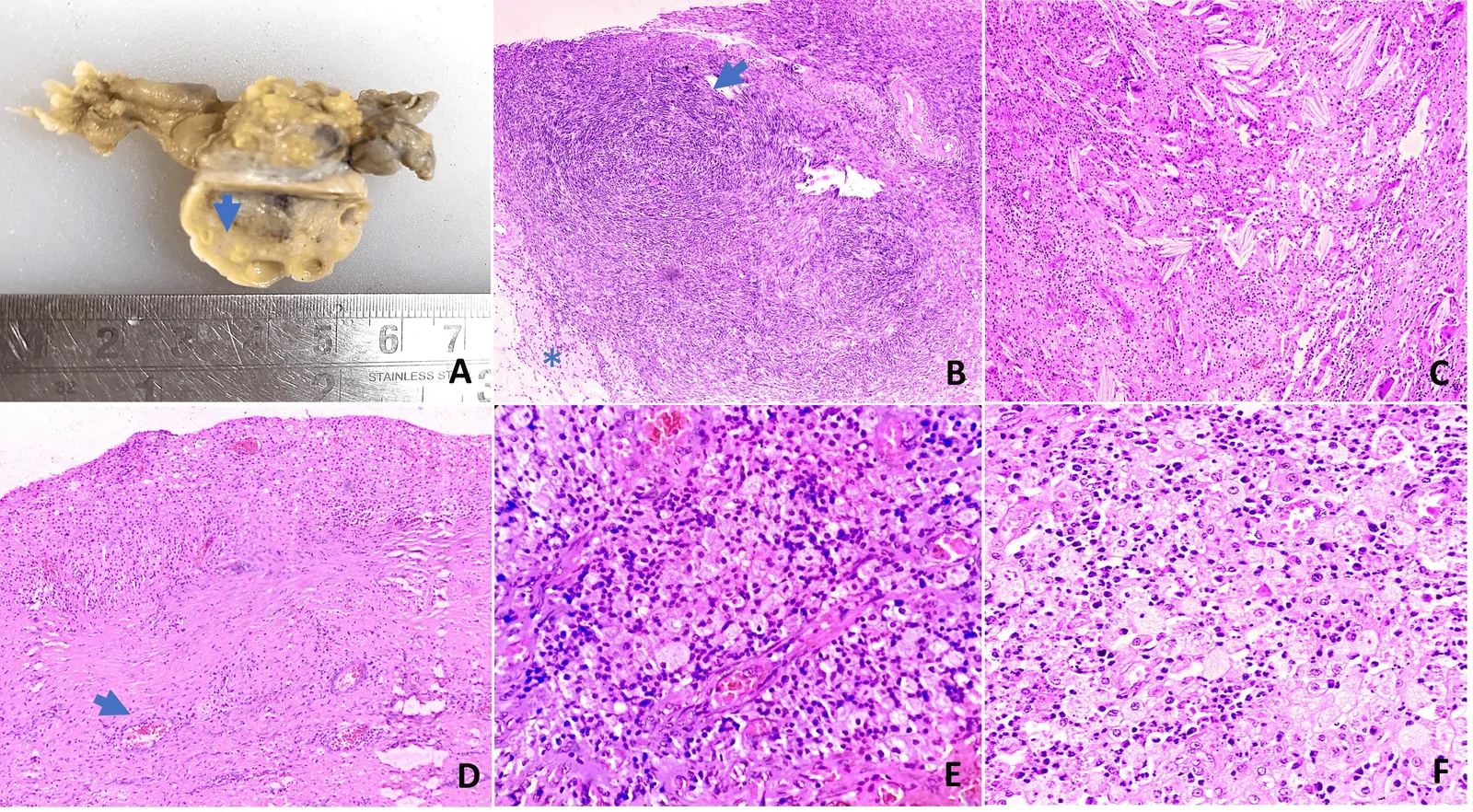
Histopathological features of xanthogranulomatous oophoritis. A-C (Case 3): A. Portion of a transected ovary specimen from a 39-year-old woman showing a solid-cystic cut surface with yellowish areas (arrow); B. Low-power view showing destruction of normal ovarian parenchyma (arrow) by sheets of foamy macrophages (asterisk) (40×, H&E); C. Cholesterol clefts flanked by scattered multinucleated giant cells and mononuclear cell infiltration (100×, H&E); D-E (Case 1): D. Proliferation of fibroblasts and neovascularization adjacent to dense surface inflammation (100×, H&E); E. High-magnification view revealing a characteristic sheet-like infiltrate dominated by abundant pale foamy histiocytes admixed with lymphocytes and plasma cells (400×, H&E); F (Case 4): In addition to histiocytes and chronic inflammation, a prominent infiltrate of neutrophils is noted (400×, H&E).

Gross pathological examination did not yield specific diagnostic findings. The majority of cases presented with a cystic ovary; although the fallopian tube was dilated in a single instance, other cases showed no macrostructural tubal abnormalities. Cut surfaces were either gray-yellow or hemorrhagic (Figure 1, 2). On microscopic evaluation, all cases demonstrated dense parenchymal infiltration within the ovary or fallopian tube, characterized by sheets of foamy (lipid-laden) macrophages intermixed with inflammatory cells. Five cases exhibited a pure chronic inflammatory infiltrate comprising lymphocytes and plasma cells, whereas two cases had a mixed inflammatory profile containing neutrophils along with chronic inflammatory cells. Multinucleated giant cells were also observed in two cases (Figure 3). Isolated ovarian involvement was seen in three cases, while the remaining four cases had isolated fallopian tube involvement. Concomitant benign lesions included leiomyomas and endometriosis; notably, none of the cases were associated with malignancy.

Detailed clinical narratives for each patient are presented below, with the key clinicopathological features summarized in Table 1.

**Table 1:** Summary of the key clinicopathological features for each case. AUB, abnormal uterine bleeding; CIN, cervical intraepithelial neoplasia; LSCS, lower segment cesarean section; TAH + BSO, total abdominal hysterectomy with bilateral salpingo-oophorectomy

S. no.	Age	Clinical presentation	Clinical diagnosis	Radiological investigation	Surgery performed	Summary of gross findings	Microscopic features	Final diagnosis	Associated risk factors
1	34	Lower abdominal pain and heaviness	Suspicious for malignancy	Complex solid-cystic pelvic mass measuring 5.2 × 4.0 × 3.4 cm, with a thick wall and multiple intramural T1-weighted hypointense nodules	Left oophorectomy	Cystic left ovarian mass measuring 5 × 5 × 1.4 cm. Cut surface showed a few gray-white to gray-yellow solid areas	Foamy macrophages ++; lymphocytes, plasma cells ++; multinucleated giant cells +	Left xanthogranulomatous oophoritis	Previous LSCS
2	55	Abdominal pain and distension	Fibroid uterus	USG: Enlarged uterus with heterogeneous, asymmetrical myometrium containing multiple well-circumscribed hypoechoic myometrial masses. Both ovaries appeared as thin-walled cystic structures without solid components or abnormal vascularity	TAH + BSO	Intramural fibroids with myometrial trabeculations. Unilocular cysts in both ovaries with a dilated left fallopian tube	Foamy macrophages ++; lymphocytes, plasma cells ++	Bilateral xanthogranulomatous salpingitis	Leiomyoma
3	39	AUB with abdominal pain	Fibroid uterus	USG: Large intramural uterine fibroid measuring 11 × 9 cm. Ovaries and fallopian tubes were normal	TAH + bilateral salpingectomy + right oophorectomy	Solid-cystic lesion in the right ovary measuring 2.4 × 2.0 × 1.7 cm	Foamy macrophages ++; lymphocytes, plasma cells ++; cholesterol clefts with giant cells +	Right xanthogranulomatous oophoritis	Leiomyoma
4	27	Acute abdominal pain	Adnexal neoplasm	USG: Intrauterine pregnancy with a large heterogeneous hypoechoic lesion measuring 13.4 × 5.7 mm posterior to the cervix and vagina, extending toward the left adnexa, with internal septations	Exploratory laparotomy with left salpingo-oophorectomy	Left ovarian multiloculated cystic mass measuring 6.5 × 3.5 × 2 cm, with a gray-yellow cut surface	Foamy macrophages ++; lymphocytes, plasma cells ++; neutrophils +	Left xanthogranulomatous oophoritis with pseudoxanthogranulomatous salpingitis	None
5	68	Postmenopausal bleeding	Incidental finding (Pap smear: CIN 1)	USG: Bulky uterus with unremarkable bilateral adnexa	TAH + BSO	No specific findings	Foamy macrophages ++; lymphocytes, plasma cells ++	Bilateral xanthogranulomatous salpingitis	Obesity
6	24	Acute abdominal pain	Endometrioma with suspicion of torsion	USG: Unilocular, avascular left-sided cyst with homogeneous ground-glass internal echoes	Salpingo-oophorectomy	Gray-brown cystic ovarian mass measuring 7 × 6.5 × 2.5 cm, filled with hemorrhagic material. Attached fallopian tube was unremarkable	Foamy macrophages ++; lymphocytes, plasma cells ++	Left xanthogranulomatous salpingitis	Endometrioma, left ovary
7	60	Lower abdominal pain and distension	Adnexal neoplasm	USG, CT scan: Posterior wall fibroid (11 × 10 mm) and a well-defined left adnexal cystic lesion measuring 4.4 × 4.1 cm with low-level internal echoes and calcific specks, suggestive of a dermoid cyst	Exploratory laparotomy with TAH + BSO	Dilated left fallopian tube measuring 2.5 × 1.5 × 1 cm and enlarged left ovary. Left ovary measured 4.5 × 2 × 1.5 cm. Cut surface was gray-white to gray-brown with focal gray-yellow areas. Lumen showed hemorrhagic material	Foamy macrophages ++; hemosiderin-laden macrophages +; lymphocytes, plasma cells ++; neutrophils ++	Left xanthogranulomatous salpingitis	Endometrioma, left ovary

Case 1

A 34-year-old multiparous (P4L4) woman presented with lower abdominal pain and heaviness for the last six months. She had a previous history of intrauterine contraceptive device (IUCD) insertion and no other significant comorbidities. Gynecological examination revealed fullness in the left fornix. USG and subsequent MRI showed a complex solid-cystic pelvic mass measuring 5.2 × 4.0 × 3.4 cm, with a thick wall and multiple intramural T1-weighted hypointense nodules, raising suspicion of an ovarian neoplasm. CA-125 levels were mildly raised (67.3 U/mL). The patient underwent left oophorectomy. On gross examination, the specimen consisted of an already opened cystic left ovarian mass measuring 5 × 5 × 1.4 cm. The cut surface showed a unilocular cyst with a few gray-white to gray-yellow solid areas. Microscopic features of xanthogranulomatous oophoritis were seen in the left ovary.

Case 2

A 55-year-old multiparous (P3L3), perimenopausal woman presented with abdominal pain and distension for the last three months. She had no significant comorbidities or previous surgical history. General physical examination was unremarkable. A per vaginal examination revealed a bulky uterus (≈12 weeks) with fullness in the bilateral fornices. Ultrasonography showed an enlarged uterus measuring 13 × 10 × 8 cm with heterogeneous, asymmetrical myometrium containing multiple well-circumscribed hypoechoic myometrial masses. Both ovaries appeared as thin-walled cystic structures without solid components or abnormal vascularity. The patient underwent total abdominal hysterectomy (TAH) with bilateral salpingo-oophorectomy (BSO). Gross examination revealed multiple intramural fibroids with myometrial trabeculations. Both ovaries showed unilocular cysts. The left fallopian tube was dilated with an adjacent paratubal cyst. Histopathology confirmed the presence of uterine leiomyoma along with concurrent adenomyosis as distinct coexisting pathologies. The ovaries showed follicular cysts, and both fallopian tubes revealed features of xanthogranulomatous salpingitis.

Case 3

A 39-year-old multiparous (P2L2) woman with hypothyroidism presented with abdominal pain and heavy menstrual bleeding with passage of clots for one year. No other comorbidity or previous surgical history was noted. Clinical examination revealed mild pallor and an enlarged uterus (≈24 weeks’ gestation). Per speculum and per vaginal examinations were unremarkable. Ultrasonography demonstrated a large intramural uterine fibroid measuring 11 × 9 cm. The patient underwent TAH with bilateral salpingectomy and right oophorectomy (TAH + bilateral salpingectomy + right oophorectomy). Intraoperatively, the uterus was markedly bulky with a large fibroid, and the right ovary appeared cystic. Gross examination of the uterus showed a large intramural fibroid, and the right ovary revealed a solid-cystic lesion measuring 2.4 × 2.0 × 1.7 cm. Both fallopian tubes were grossly unremarkable. Microscopy confirmed leiomyoma and showed features of XGI in the right ovary, while the fallopian tubes were unremarkable.

Case 4

A 27-year-old woman, primigravida at 25 weeks of gestation (P1), presented with episodes of severe lower abdominal pain during the last 15 days. She had no significant medical comorbidities or history of previous surgery. Clinical examination revealed per-abdominal findings corresponding to 24 weeks of gestation. Ultrasonography performed for routine fetal well-being showed a gravid uterus with a single intrauterine pregnancy at a gestational age of 24 weeks. In addition, it revealed a large heterogeneous hypoechoic lesion measuring 13.4 × 5.7 mm posterior to the cervix and vagina, extending toward the left adnexa, with internal septations, raising the possibility of an adnexal neoplasm. CA-125 levels were within normal limits, while alpha-fetoprotein levels were mildly raised. At this point, the patient underwent exploratory laparotomy with left salpingo-oophorectomy. Gross examination revealed a left ovarian multiloculated cystic mass measuring 6.5 × 3.5 × 2 cm. The cut surface showed gray-yellow areas. A histopathological diagnosis of xanthogranulomatous oophoritis with pseudo-xanthogranulomatous salpingitis was rendered.

Case 5

A 68-year-old postmenopausal obese woman (P3L2A1), hypertensive and on medications, presented with two episodes of postmenopausal bleeding during the last three months and discharge per vaginum for five years. No other comorbidities or previous surgical history was noted. The gynecological examination was unremarkable. Pap smear was reported as CIN 1. Ultrasonography revealed a bulky uterus, while the bilateral adnexa appeared unremarkable. The patient underwent TAH with bilateral salpingo-oophorectomy (TAH + BSO). Intraoperatively, the uterus was bulky, whereas both fallopian tubes and ovaries appeared grossly normal. Gross examination of the uterus, cervix, and bilateral adnexa did not reveal any significant abnormality. Microscopic evaluation showed features of bilateral xanthogranulomatous salpingitis and a corpus luteal cyst in the ovary.

Case 6

A 24-year-old married woman (P1L1) with a known history of hypothyroidism presented with sudden-onset severe abdominal pain. She had a history of thyroidectomy 10 years previously; however, no records were available. She had no other comorbidities. Clinical examination revealed a distended, tender, rigid abdomen with guarding. Per speculum and per vaginal examinations were unremarkable. Ultrasonography revealed a unilocular, avascular left-sided cyst with homogeneous ground-glass internal echoes, suggestive of an endometrioma. Salpingo-oophorectomy was performed, and the specimen was sent for histopathological analysis.

Gross examination revealed an already opened gray-brown cystic ovarian mass measuring 7 × 6.5 × 2.5 cm. The cut surface was gray-brown to gray-tan and filled with hemorrhagic material. The attached fallopian tube was grossly unremarkable. Microscopy showed an endometriotic cyst of the ovary with XGI in the fallopian tube.

Case 7

A 60-year-old postmenopausal diabetic woman (P3L3) presented with a history of lower abdominal pain and distension of two months’ duration. A previous history of tubal ligation was noted 25 years previously. She had no other comorbidity. Gynecological examination showed a healthy cervix, and the bilateral fornices were clear. Ultrasonography revealed a postmenopausal uterus with a small posterior wall fibroid (11 × 10 mm) and a well-defined left adnexal cystic lesion measuring 4.4 × 4.1 cm with low-level internal echoes and calcific specks, suggestive of a dermoid cyst. Contrast-enhanced computed tomography demonstrated a well-defined, thick-walled cystic lesion measuring 5.2 × 4.8 cm, suspicious for a neoplastic etiology. The patient was planned for exploratory laparotomy with TAH, bilateral salpingo-oophorectomy (TAH + BSO), and omentectomy.

Intraoperatively, a 5 × 5 cm pelvic mass was identified, inseparable from the uterus. The left ovary could not be identified separately, and approximately 20 mL of pus was drained from the pelvic cavity. Culture sensitivity of the pus showed streptococcal infection. Gross examination showed two intramural fibroids with a dilated left fallopian tube measuring 2.5 × 1.5 × 1 cm. The cut surface showed gray-brown to focal gray-yellow areas. The left ovary measured 4.5 × 2 × 1.5 cm and showed hemorrhagic material. Microscopic examination revealed features of xanthogranulomatous salpingitis with an endometriotic cyst of the left ovary.

## Discussion

XGSO represents a rare and specialized form of tissue reaction characterized by chronic destructive inflammation. Initially documented in the female genital tract by Gray and Libbey in 2001, it remains an exceptionally rare clinical entity with limited data published in the literature [2]. While XGI typically involves the gallbladder and kidney, its manifestation within the ovaries and fallopian tubes is a significant diagnostic challenge. The clinical hallmark of XGSO is its remarkably heterogeneous presentation, which frequently masquerades as advanced ovarian malignancy or refractory PID. The formation of complex solid-cystic pelvic masses coupled with dense adhesions to the neighboring pelvic organs mimics local invasion seen in advanced carcinomas [1,3-5]. This overlapping clinical presentation, along with nonspecific elevation of tumor markers like CA-125, frequently results in preoperative diagnostic errors and drives surgical overdiagnosis.

The exact pathophysiological mechanisms underlying this entity remain poorly understood, but multiple risk factors have been implicated. As previously stated in the literature, chronic bacterial infection is a primary driver, with common uropathogens and pyogenic bacteria such as *Escherichia coli*, streptococci, and *Proteus mirabilis*. Ineffective bacterial clearance by phagocytes leads to persistent microabscesses that instigate tissue necrosis and subsequent macrophage phagocytosis of the liberated cellular lipids. The fact that these organisms are frequently cultured from these xanthogranulomatous lesions strongly supports an infectious origin [9,10]. Mechanical luminal obstruction secondary to prior surgeries (such as LSCS) or tubal ligations further promotes stasis. Further, coexisting pelvic pathologies such as endometriosis and leiomyomas, or long-term use of IUCDs, play a crucial role by inducing chronic microhemorrhage and cyclical tissue destruction, generating abundant cellular debris and local lipid accumulation [7,9-15]. In this series, patients also had risk factors such as previous surgeries or associated endometriosis, leiomyomas, and PID (Table 1).

Very rarely, XGI involving the female genital tract is seen in pregnant females, with only a few cases reported in the literature to date [16,17]. XGSO occurring during pregnancy presents a critical diagnostic and therapeutic challenge. In a pregnant patient, ultrasound and MRI frequently reveal a complex, heterogeneous solid-cystic pelvic mass that raises immediate suspicion for epithelial ovarian cancer or an aggressive germ cell tumor. This diagnostic dilemma is further compounded by overlapping clinical symptoms, such as lower abdominal pain and mild fever, and occasionally elevated tumor markers like CA-125, which can naturally fluctuate during gestation. Management strategies during pregnancy require careful individualization to balance maternal health with fetal safety. Since a definitive diagnosis relies entirely on histopathological examination, initial treatment involves surgical intervention, such as exploratory laparotomy or laparoscopy with unilateral oophorectomy or cystectomy. In one such case that we reported, a female at 24 weeks of gestation presented with clinical and radiological features that simulated a neoplasm. She underwent a selective oophorectomy, successfully managing the condition while preserving fetal health.

The primary differential diagnosis includes pseudo-xanthomatous salpingitis (PXS), malakoplakia, and other specific granulomatous infections. PXS represents a key differential diagnosis and is heavily linked to endometriosis but is distinguished microscopically by the presence of pigment-laden (hemosiderin) macrophages and lack of acute or chronic inflammatory cell infiltration [18,19]. Malakoplakia shares a similar histiocytic appearance but can be ruled out by the total absence of Michaelis-Gutmann bodies, which are basophilic, concentric, calcified cytoplasmic inclusions within the histiocytes. Systemic infections like tuberculosis or fungal infections with a granulomatous picture on histology need to be excluded carefully. Tuberculous salpingo-oophoritis is distinguished by well-formed epithelioid granulomas with caseous necrosis, which can be definitively confirmed using Ziehl-Neelsen (acid-fast bacilli) ancillary stains and mycobacterial cultures. Fungal infections can similarly be identified by utilizing special histochemical stains like periodic acid-Schiff or Grocott’s methenamine silver to visualize fungal hyphae or yeast forms [9,11,20].

This study is limited by its retrospective single-center design and relatively small sample size. Microbiological correlation and ancillary investigations were not available for all the patients. The findings may not be generalizable because of the rarity of this entity. Larger multicenter studies are required to further define the clinicopathological spectrum and optimize treatment options.

## Conclusions

XGSO can occur as a primary lesion itself or in association with other primary lesions of the female genital tract, resulting in varied clinical presentations. Histopathological analysis is essential for accurate identification and differentiation from neoplasm or other granulomatous conditions. Increasing clinical awareness of this entity is vital to optimize intraoperative decision-making, encourage the use of frozen sections, and avoid unnecessary radical surgeries in benign PIDs.
